# Distributed Multi-Agent Deep Reinforcement Learning-Based Transmit Power Control in Cellular Networks

**DOI:** 10.3390/s25134017

**Published:** 2025-06-27

**Authors:** Hun Kim, Jaewoo So

**Affiliations:** Department of Electronic Engineering, Sogang University, Seoul 04107, Republic of Korea; hunkim@sogang.ac.kr

**Keywords:** multi-cell network, multi-agent deep reinforcement learning, centralized training with decentralized execution, transmit power control

## Abstract

In a multi-cell network, interference management between adjacent cells is a key factor that determines the performance of the entire cellular network. In particular, in order to control inter-cell interference while providing a high data rate to users, it is very important for the base station (BS) of each cell to appropriately control the transmit power in the downlink. However, as the number of cells increases, controlling the downlink transmit power at the BS becomes increasingly difficult. In this paper, we propose a multi-agent deep reinforcement learning (MADRL)-based transmit power control scheme to maximize the sum rate in multi-cell networks. In particular, the proposed scheme incorporates a long short-term memory (LSTM) architecture into the MADRL scheme to retain state information across time slots and to use that information for subsequent action decisions, thereby improving the sum rate performance. In the proposed scheme, the agent of each BS uses only its local channel state information; consequently, it does not need to receive signal messages from adjacent agents. The simulation results show that the proposed scheme outperforms the existing MADRL scheme by reducing the amount of signal messages exchanged between links and improving the sum rate.

## 1. Introduction

As the number of users requiring high-data-rate services in cellular networks rises, the cell radius becomes smaller, increasing the importance of managing interference between cells [1,2,3]. In multi-cell networks, effective interference management is crucial for maintaining signal quality, maximizing spectral efficiency, ensuring a reliable quality of service (QoS), and expanding network capacity [4]. The transmit power of a base station (BS) not only reaches the intended user but also creates unintended interference with other users in other cells. Hence, effective transmit power control not only improves data rates by delivering stronger signals to intended users but also improves overall spectral efficiency by mitigating inter-cell interference with other users [5]. Hence, optimizing the transmit power of BSs is essential to maintain signal quality, mitigate interference, and maximize spectral efficiency. Such intelligent transmit power control strategies enhance overall system capacity, improve user QoS, and achieve fair radio resource control across cells [6]. However, with the addition of more BSs, controlling the transmit power of each BS in a multi-cell network becomes an increasingly difficult problem.

Recently, an increasing number of studies have applied reinforcement learning (RL) to solve the power control problem in wireless networks [7,8,9]. RL is a branch of machine learning (ML) in which an agent interacts with an environment and learns, through trial and error, a behavioral strategy that maximizes reward [10]. Because RL can capture long-term dependencies and rapidly adapt to dynamically changing wireless environments, it has seen significant use in solving transmit power control problems [11]. Deep reinforcement learning (DRL), which integrates deep neural network (DNN) with RL, effectively models state–action relationships in complex, high-dimensional environments, making it suitable for even more challenging wireless environments [12]. However, solving the power control problem using RL or DRL for interacting BSs in a multi-cell network can be challenging because it requires considering both the dynamic nature of the wireless environment and the interactions between BSs [13]. Consequently, many researchers have introduced multi-agent deep reinforcement learning (MADRL) to solve the power control problem in multi-cell networks. In MADRL, each agent learns an optimal policy in dynamic environments by cooperating or competing with each other [12,14].

The transmit power control problem is inherently non-convex and computationally complex, and the challenge becomes more severe as the network size increases. Researchers have integrated techniques from geometric programming, game theory, information theory, and Pareto theory into multi-agent algorithms to address these issues [15]. These integrated approaches aim to maximize the system capacity and scalability of large-scale networks by supporting distributed decision-making and have prompted investigations into various MADRL-based transmit power control strategies, including centralized, distributed, and decentralized learning methods [8,16]. Centralized methods leverage complete, real-time state information to achieve superior performance, but each agent transmits their state information to a central server, which incurs significant signaling overhead and increases the complexity of the central server. In contrast, distributed and decentralized learning methods limit each agent’s observation to a portion of the environment, thereby reducing the signaling overhead; however, this approach may result in slower convergence and lower model accuracy [17]. To overcome these challenges, researchers have widely adopted the centralized training with decentralized execution (CTDE) framework in power control studies [18,19]. Researchers have leveraged the CTDE framework, which combines the benefits of centralized training and decentralized execution, to effectively model multi-agent systems. They have widely applied this approach to solve various wireless radio resource management (RRM) problems, such as the problem of controlling the BS’s transmit power [20,21]. In recent wireless resource management research, long short-term memory (LSTM) has been actively studied to effectively handle time-varying wireless environments [22,23,24,25]. LSTM, a variant of recurrent neural networks (RNNs), can learn long-term data dependencies more accurately than traditional DNNs or convolutional neural networks (CNNs), and can efficiently model the interrelationship between past and future states through a sequence-oriented structure. Recent researchers have been conducting research combining LSTM and RL to leverage the long-term dependency and predictive excellence of LSTM in time-series data [24,25].

The contributions of this paper are as follows. First, we propose a novel LSTM-based multi-agent actor–critic (MAAC) network to control the transmit power of each BS in multi-cell networks, thereby aiming to maximize the sum rate. Second, the proposed scheme uses only local channel state information (CSI), which dramatically reduces the signaling overhead caused by signaling messages used to exchange information between agents. Third, the simulation results show that the proposed scheme outperforms conventional deep Q-network (DQN)-based or QMIX-based MADRL schemes. In particular, the DQN-based MADRL scheme requires the signaling messages to exchange information between agents, which results in significant signaling overhead, whereas the proposed scheme does not require information from other agents.

## 2. Related Works

Researchers have extensively studied transmit power control in cellular networks using a wide range of approaches, including model-based optimization techniques, data-driven methods, and RL. For instance, a weighted minimum mean square error (WMMSE)-based scheme transforms the sum rate maximization problem into a weighted least mean square error problem for each user or transmitter–receiver pair and iteratively solves the resulting convex subproblems to find a solution to the original non-convex problem [26]. Similarly, fractional programming (FP) addresses an optimization problem with the objective expressed as the ratios of functions by employing techniques such as the Dinkelbach method and quadratic transform [27]. Although these model-driven optimization techniques perform powerfully, they encounter high computational complexity, sensitivity to initial conditions, and poor scalability because they rely on the instantaneous global CSI of all receivers.

To overcome the above problems of model-based optimization techniques, researchers have recently proposed data-based methods for transmit power control in wireless networks. For example, the authors of [28] proposed a method for training a DNN that allocates transmit power to reused channels to suppress interference. However, this approach relies on the quality of existing model-based optimization techniques and has limitations such as high offline computational costs and difficulty in adapting to dynamic environmental changes. The authors of [29] introduced a CNN-based distributed processing method to solve the transmit power control problem, aiming to reduce signaling overhead due to signal message exchanges and to support real-time processing. However, since the CNN is originally optimized for image processing, it may be limited in terms of scalability and generalization in complex multi-cell network environments. The authors of [30] proposed a scalable graph neural network (GNN)-based architecture for transmit power control and beamforming optimization that satisfies permutation equivalence, provides high computational efficiency, and generalizes well to large-scale problems. However, these methods rely on data that may not accurately represent real-world conditions, making the tracking of changes over time difficult and incurring high training data costs.

To address the limitations of model-based optimization techniques and data-based methods, several studies have proposed RL-based approaches for RRM and interference management in various wireless networks [31,32,33]. The authors of [31] proposed a distributed resource control scheme based on DRL that determines the resource blocks and transmit power of vehicle-to-vehicle (V2V) links using a DQN in an environment where V2V networks and vehicle-to-infrastructure (V2I) networks share radio resources and cause interference, reducing power consumption and delay while maximizing the sum of V2I and V2V links. The authors of [32] proposed an RL-based joint beamforming scheme for V2I systems that considers alignment overhead and optimizes antenna beamwidths and beam alignment spacing by leveraging past and future compensation, thereby achieving better average throughput and link stability compared with existing approaches. The authors of [33] proposed an RL-based joint power control and channel allocation scheme that utilizes statistical CSI to address severe interference caused by high-density access points in dense wireless local area network environments. Based on the correlation between transmit power and channel, the researchers derived the optimal joint optimization strategy through offline Q-learning.

A practical cellular network consists of multiple cells, where the cells interfere with each other. Hence, the transmit power control based on RL should take the inter-cell interference in multi-cell networks into consideration. Accordingly, research based on the MADRL or CTDE framework is actively being conducted to effectively model the characteristics of multi-cell networks and to improve performance [34]. The authors of [34] used a DQN network parameter-sharing method based on a single meta-agent approach to solve the problem of controlling the transmit power of a BS in a multi-cell network. However, this method may incur additional signal message overhead because parameter-sharing between agents also occurs during the inference phase of the model. The authors of [19] used the QMIX-based MADRL scheme to control the transmit power of BSs in multi-cell networks. This approach significantly reduces the signal message overhead because each BS performs transmit power control using only local CSI, eliminating the need to share CSI with neighboring BSs. The authors of [25] examined a dynamic multi-channel access scenario in which users connect to a single channel at each time slot and randomly join and leave. To improve throughput and fairness among active users, this study proposes a distributed multi-agent reinforcement learning (MARL) approach that deterministically selects channel access policies over multiple consecutive time slots and employs LSTM to continuously track changes in user states and traffic over time. This approach supports effective policy learning and adaptation in wireless environments because it captures strong temporal dependencies.

The remainder of this paper is organized as follows: Section 2 reviews the related work and its limitations. Section 3 describes the system model. Section 4 details the proposed scheme, including the states, actions, and rewards used in MADRL. Section 5 presents the simulation results, and Section 6 concludes this paper. For the sake of clarity, the main symbols and their descriptions used in this paper are summarized in Table 1.

## 3. System Model

**Figure 1:**
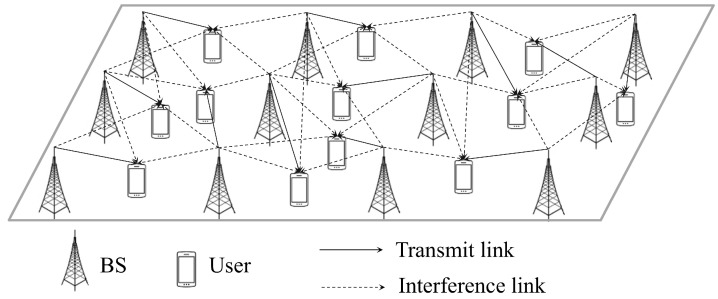
A system model.

We consider a downlink communication scenario in a multi-cell network comprising multiple BSs and multiple users, as shown in Figure 1. This type of network scenario has been extensively used to model wireless ad hoc networks [35] and is equally applicable for modeling multi-cell wireless networks [34]. The set of BS indices is denoted by K={1,2,…,K}, where *K* is the total number of BSs in the multi-cell network. Each user is assumed to be served by a single BS, i.e., each user has one transmit link and multiple interference links, where the user served by BS *k* is denoted by $u_{k}$. Each BS and user has a single antenna. Each BS is located at the center of a cell, while users are randomly distributed within the boundaries of that cell.

The system operates as a fully synchronized time-slotted network with a fixed slot duration *T*. Each cell uses the same frequency band, making inter-cell interference a critical issue. We adopt a block-fading model, which assumes that the wireless channel remains constant during each time slot and changes independently between slots. Specifically, the downlink channel gain from BS *k* to user *j* at time slot *t* is modeled as follows [34]:

(1) $$\begin{array}{c} g_{k,j}^{t}={\left|h_{k,j}^{t}\right|}^{2}\;\beta_{k,j}^{t},\;t=1,2,\ldots , \end{array}$$

where $h_{k,j}^{t}$ represents the small-scale Rayleigh fading component, and $\beta_{k,j}^{t}$ represents the large-scale fading component, which includes both path loss and log-normal shadowing. |·| is the modulus operation. The small-scale fading $h_{k,j}^{t}$ is modeled using Jake’s fading model as a first-order complex Gauss–Markov process as follows [36]:

(2) $$\begin{array}{c} h_{k,j}^{t+1}=\rho \;h_{k,j}^{t}+\sqrt{1-\rho^{2}}\;e_{k,j}, \end{array}$$

where $e_{k,j}$ are independent and identically distributed (i.i.d.) circularly symmetric complex Gaussian (CSCG) random variables with unit variance and the correlation coefficient is given by $\rho =J_{0}\left(2\pi f_{d}T\right)$, with $J_{0}\left(\cdot \right)$ being the zeroth-order Bessel function of the first kind. *T* and $f_{d}$ are the slot duration and the maximum Doppler frequency, respectively. The large-scale fading component $\beta_{k,j}^{t}$ is modeled as follows:

(3) $$\begin{array}{c} \beta_{k,j}^{t}=PL\left(d\right)+X_{\alpha }^{t}, \end{array}$$

where *d* is the distance between BS *k* and user *j*, and PL(d) is the distance-based path loss derived from the 3GPP TR 25.942 standard [37]. The term $X_{\alpha }^{t}$ represents log-normal shadowing, which remains nearly constant over many time slots. The shadowing process is updated as follows:

(4) $$\begin{array}{c} X_{\alpha }^{t}=X_{\alpha }^{t-1}+\sigma_{s}\;e_{k,j}^{\mathrm{shad}}, \end{array}$$

where $\sigma_{s}$ denotes the log-normal shadowing standard deviation, $X_{\alpha }^{0}$ is drawn from $N(0,\sigma_{s}^{2})$, and $e_{k,j}^{\mathrm{shad}}$ are i.i.d. Gaussian random variables with unit variance. The downlink transmit power vector of BSs at time slot *t* is denoted by

(5) $$\begin{array}{c} p^{t}=\left[p_{1}^{t},p_{2}^{t},\ldots ,p_{K}^{t}\right], \end{array}$$

where $p_{k}^{t}$ is the transmit power of BS *k* at time slot *t*. The signal-to-interference-plus-noise ratio (SINR) received by user *k* in time slot *t* can then be expressed as follows:

(6) $$\begin{array}{c} \gamma_{k}^{t}\left(p\right)=\frac{g_{k,k}^{t}\;p_{k}^{t}}{\sum_{i\neq k}g_{i,k}^{t}\;p_{i}^{t}+\sigma^{2}},\;k\in K, \end{array}$$

where $\sigma^{2}$ is the additive white Gaussian noise (AWGN) power spectral density (PSD). The downlink spectral efficiency of user *k* at time slot *t* is given by

(7) $$\begin{array}{c} C_{k}^{t}\left(p\right)=\log_{2}\left(1+\gamma_{k}^{t}\left(p\right)\right),\;k\in K. \end{array}$$

At each time slot *t*, the transmit power control problem is the maximization of the sum rate, as follows:

(8) $$\begin{array}{cc} \max_{p} & \sum_{k=1}^{K}C_{k}^{t}\left(p\right), \end{array}$$

(9) $$\begin{array}{ccc} \mathrm{subject}\mathrm{to} & 0\leq p_{k}\leq P_{\max}, & \forall k\in K, \end{array}$$

where $P_{max}$ is the maximum power of each BS. However, finding the optimal parameter p of the transmit power vector is known to be an NP-hard problem [38].

## 4. Proposed LSTM-Based MAAC Network for Transmit Power Control

### 4.1. Typical MADRL Network

**Figure 2:**
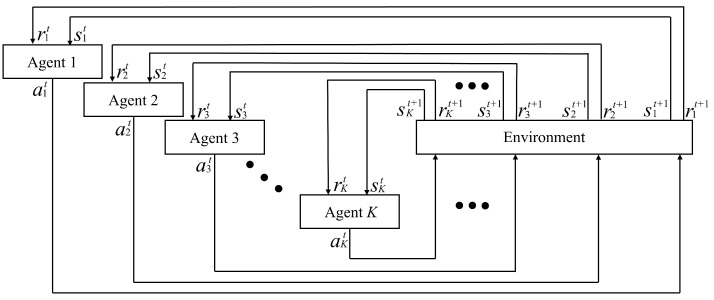
A typical MARL structure.

Conventional RL uses a single agent to maximize long-term rewards based on environment observations, and therefore, optimizing the individual rewards of multiple devices with different characteristics is difficult. In contrast, MARL tries to enhance the overall system performance by taking each device’s unique properties into consideration. Specifically, MADRL extends a single-agent RL with DNNs that enable cooperation or competition among agents in complex environments. Figure 2 shows a typical structure of the MARL framework [39]. Each agent (agent 1, agent 2, …, agent *K*) observes its own state $s_{k}^{t}$, selects an action $a_{k}^{t}$ based on its policy, and then receives a corresponding reward $r_{k}^{t}$ from the environment. The collective actions of all agents affect the environment, which updates the state and reward for the next time step, t+1.

**Figure 3:**
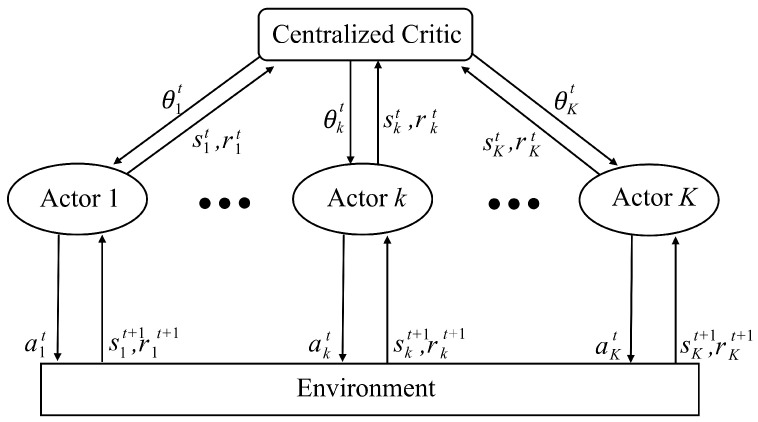
A typical CTDE-based MARL structure.

Multi-agent systems (MASs) are distributed systems where autonomous agents interact to achieve a common goal, each making decisions based solely on its local observations [40]. However, individual agents often cannot fully capture the global state, making traditional Markov decision process (MDP) assumptions hard to satisfy and necessitating more complex models like partially observable Markov decision processes (POMDPs) or Markov games [41]. Moreover, the joint action space expands exponentially with the number of agents, complicating the evaluation of each agent’s impact on overall performance. To address these challenges, the CTDE framework is widely adopted [17,18,19]. In CTDE, a centralized critic evaluates the joint state–action value using local information from all agents during training, and during execution, each agent makes real-time decisions based solely on its local observations. This approach combines the benefits of robust, centralized learning with the efficiency of decentralized execution. Figure 3 illustrates a CTDE structure, where each actor (actor 1, actor 2, …, actor *K*) independently interacts with the environment by selecting actions based on its local state and receiving rewards.

The decentralized POMDP can be expressed as a tuple {K,S,A,P,R,Ω,γ} [41]. Here, K is the set of agent indices, which are regarded as the distributed BSs in this paper; S is the global state space, which contains local observations of all agents; and A is the global action space such that $A=\times_{k}A_{k}$, where × is the Cartesian product operator. $P\left(s^{t+1}|a^{t},s^{t}\right)$ is the probability of transitioning into a state $s^{t+1}\in S$ by taking action $a^{t}\in A$ at state $s^{t}\in S$. At time *t*, $s^{t}\in S$ and $a^{t}\in A$ denote the global state and action, respectively. R is the reward function for being in state $s^{t}\in S$ when taking action $a^{t}\in A$, and all agents might share the same reward function. $\Omega_{k}$ denotes the local observation of agent *k*, and the joint observation space is given by $\Omega =\times_{k}\Omega_{k}$. γ is the discount factor, γ∈[0,1], which determines the present value of future rewards.

### 4.2. Proposed MAAC Network

In the MAS described in this paper, for all k∈K, BS *k* is considered identical to agent *k*. That is, each BS is treated as an independent agent within the MADRL, and the two terms can be used interchangeably. Through this perspective, by analyzing the decision-making and interaction of the BS as the behavior of the agents, the MADRL can be effectively applied to the transmit power control problem. We define the local state at agent *k* in time slot *t* as follows:

(10) $$\begin{array}{c} s_{k}^{t}=\left(g_{k,k}^{t},\;E_{k}^{t}\right), \end{array}$$

where $g_{k,k}^{t}$ represents the instantaneous channel gain between users associated with BS *k* at time slot *t*. $E_{k}^{t}$ denotes the interference-plus-noise power observed by the user served by BS *k*, where the interference is estimated based on the previously used transmit powers of other BSs, combined with the noise power. The $E_{k}^{t}$ can then be expressed as follows:

(11) $$\begin{array}{c} E_{k}^{t}=\sum_{j\neq k}\left(g_{j,k}^{t}\;p_{j}^{t-1}\right)+\sigma^{2}. \end{array}$$

At the beginning of time slot *t*, each user calculates the instantaneous interference by combining the transmit power in the time slot (t−1) with the channel gains measured in the current time slot *t* [19]. After adding the instantaneous interference and the noise, each user produces the interference-plus-noise, which is used as the input of the RL agent.

In MADRL, reducing the state feature simplifies learning by decreasing the size of the neural network’s input layer. In MADRL, reducing state features reduces the dimensionality of the input layer, which reduces unnecessary noise that may occur during model learning, improves learning stability and generalization performance, and ultimately helps the agent find the optimal policy more effectively. The global state based on the local state can be expressed as follows:

(12) $$\begin{array}{c} s^{t}=\left\{s_{1}^{t},\ldots ,s_{k}^{t},\ldots ,s_{K}^{t}\right\}. \end{array}$$

Agent *k* estimates or computes $s_{k}$ based on local observations $o_{k}$. Agent *k* uses the pilot signal or interference level of $o_{k}$ to determine or update $s_{k}$. We define the action of each agent as the transmit power. With local state $s_{k}^{t}$, agent *k* selects an action $a_{k}^{t}\in a$. The current policy $\mu_{k}\left(a_{k}^{t}|s_{k}^{t}\right)\in \left[0,1\right]$ represents the probability of action $a_{k}^{t}$ being selected under local state $s_{k}^{t}$. We define the global action as follows:

(13) $$\begin{array}{c} a^{t}=\left\{a_{1}^{t},\ldots ,a_{k}^{t},\ldots ,a_{K}^{t}\right\}. \end{array}$$

After all agents simultaneously select actions, the environment provides a reward according to the reward function $R(s^{t},a^{t})$. We define the reward function as follows:

(14) $$\begin{array}{c} R\left(s^{t},a^{t}\right)=\omega \sum_{k=1}^{K}C_{k}^{t},\left(0<\omega \leq 1\right), \end{array}$$

where ω is a weighting factor. In the learning process of MADRL, if the scale of the reward is too large, the result does not converge. To prevent this lack of convergence, the scale of the reward is adjusted through a weighting factor. The reward function is defined as the weighted sum of the spectral efficiencies of all users. The summation $\sum_{k=1}^{K}C_{k}^{t}$ aggregates the spectral efficiencies of all users. This reward structure promotes cooperative behavior between agents, accelerating and stabilizing the learning process, ultimately enabling more efficient transmit power control in the entire network.

The proposed MAS is based on the DDPG approach, a model-free deterministic actor–critic method designed for continuous action spaces. In this framework, the actor directly outputs an action value, and the critic evaluates the state–action pair. Target networks are updated via soft updates to stabilize training, and experience replay is used to reduce data correlations and to ensure more stable learning. The critic network takes the states of all agents $s=(s_{1},\ldots ,s_{k},\ldots s_{K})$ and the actions of all agents $a=(a_{1},\ldots ,a_{k},\ldots a_{K})$ as the input and computes the Q-values Q(s,a;ϕ), where ϕ represents the parameters of the critic network. Each agent’s target actor network generates the action $a^{\prime }=\left(a_{1}^{\prime },\ldots ,a_{k}^{\prime },\ldots a_{K}^{\prime }\right)$ for the next state $s^{\prime }$, and the target critic network computes the target Q-value as follows:

(15) $$\begin{array}{c} y=r+\gamma \;Q_{\mathrm{target}}\left(s^{\prime },a^{\prime };\phi^{\prime }\right), \end{array}$$

where r is the reward vector that may include individual rewards for each agent. γ is the discount factor, and $\phi^{\prime }$ denotes the parameters of the target critic network. The critic network is updated to minimize the mean squared error (MSE) between the current Q-value and the target Q-value as follows:

(16) $$\begin{array}{c} L\left(\phi \right)=E_{(s,a,r,s^{\prime })∼B}\left[{\left(Q\left(s,a;\phi \right)-y\right)}^{2}\right], \end{array}$$

where *B* represents the distribution of experiences sampled from the replay buffer. Each agent *k*′s actor network $\mu_{k}\left(s_{k};\theta_{k}\right)$ selects an action based on its local observation $s_{k}$. Each agent updates its policy to maximize the Q-value evaluated by the critic network as follows:

(17) $$\begin{array}{c} L\left(\theta_{k}\right)=-E_{s∼B}\left[Q\left(s,a_{1},\ldots ,a_{k},\ldots a_{K};\phi \right)\right]. \end{array}$$

The joint action composed of all agents’ actor outputs is updated in a way that maximizes the critic’s Q-value evaluation. For agent *k*, whose expected return is denoted by $E\left[R_{k}\right]$, the deterministic policy gradient theorem states that the gradient of its expected cumulative reward, $J\left(\theta_{k}\right)=E\left[R_{k}\right]$, is as follows:

(18) $$\begin{array}{c} \nabla_{\theta_{k}}J\left(\theta_{k}\right)=E_{s∼B}\left[\nabla_{\theta_{k}}\mu_{k}\left(s_{k};\theta_{k}\right)\;\nabla_{a_{k}}Q\left(s,a_{1},\ldots ,a_{k},\ldots a_{K};\phi \right)\;{|}_{a_{k}=\mu_{k}\left(s_{k};\theta_{k}\right)}\right]. \end{array}$$

When each agent *k* updates its policy $\mu_{k}$, it first calculates how its action $a_{k}$ affects the Q-function using $\nabla_{a_{k}}Q$. Since the actor network $\mu_{k}$ determines $a_{k}$, we then apply the chain rule to combine $\nabla_{a_{k}}Q$ with $\nabla_{\theta_{k}}\mu_{k}$ in order to compute the gradient with respect to $\theta_{k}$.

**Figure 4:**
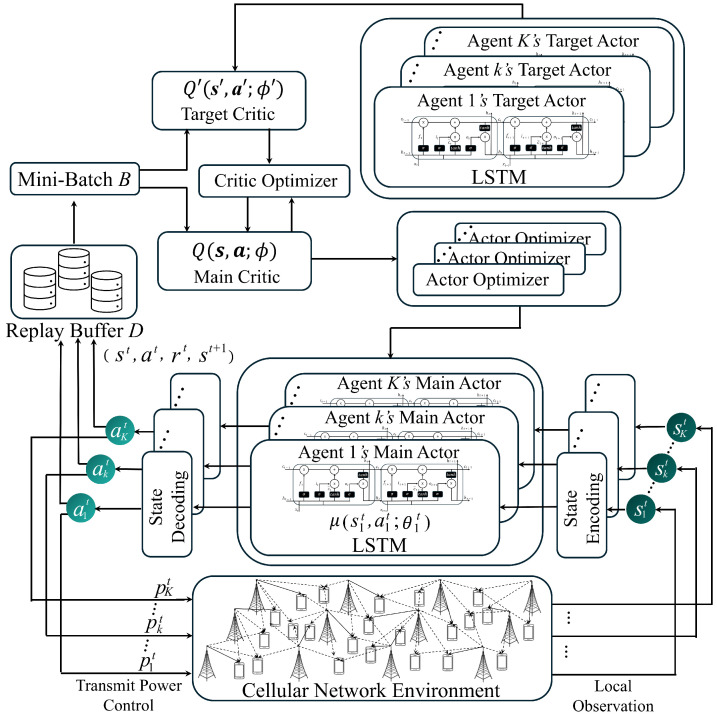
A proposed LSTM-based MAAC network.

Figure 4 shows the proposed MADRL network to determine the transmit power of BSs in the multi-cell network. The main components of the actor network are an encoder, a forward LSTM, and a decoder. First, the encoder maps the local state to a hidden representation using a fully connected layer followed by ReLU activation. Next, the LSTM processes these encoded features to capture temporal dependencies. Finally, the decoder refines the LSTM output using batch normalization and LeakyReLU and then passes it through the final fully connected layer using sigmoid activation to produce a transmit power value that is bounded by [0,1]. The critic network has a similar structure, consisting of an encoder, an LSTM, and a decoder that use the same activation functions. However, the critic takes the concatenated states and actions of all agents as the input and the Q-value for each agent’s state–action pair as the output.

The proposed network includes the LSTM to continuously track state changes over time and preserve historical observation data. The DRL combined with the LSTM has been shown to have better convergence speed and performance than the traditional DQN algorithm [24]. Moreover, the LSTM addresses the weakness of neural networks in processing long-term memory information and has excellent prediction performance in time-series wireless environments. Each LSTM gate helps reduce the influence of unnecessary past information during training. As a result, the final output generates a transmit power value in the range [0,1], which is used to update the LSTM cell state/gates. This mechanism allows the model to autonomously summarize and utilize important state information without separately inputting the transmit power of the previous time slot.

In the proposed LSTM-based MAAC network, the main and target actor parameters are updated as follows:

(19) $$\begin{array}{c} \theta^{\prime }=\left(1-\tau \right)\theta^{\prime }+\tau \theta ,\;\phi^{\prime }=\left(1-\tau \right)\phi^{\prime }+\tau \phi , \end{array}$$

where τ is the soft update parameter; θ and ϕ represent the current parameters of the actor and critic networks, respectively; and $\theta^{\prime }$ and $\phi^{\prime }$ are the parameters of the corresponding target network. This strategy gradually blends new parameters into the target networks, ensuring consistent representation and more stable training. The critic optimizer is responsible for updating the parameters of the critic network by minimizing the MSE between the current Q-values and the target Q-values. That is, it performs gradient descent on the critic loss so that the critic network can accurately estimate the value of state–action pairs. On the other hand, the actor optimizer updates the parameters of each agent’s actor network by minimizing the negative Q-value output expected by the critic network. This effectively maximizes the estimated Q-value for the actions chosen by the actor, guiding each agent to select actions that are expected to yield higher rewards. Hence, while the critic optimizer focuses on learning accurate value estimates, the actor optimizer enhances the policy by driving action selection toward those that maximize future rewards.

The total number of parameters for the actor network is calculated by summing the contributions from each component. In particular, let *I* denote the input dimension, *H* denote the hidden dimension, and *A* denote the output dimension. The encoder projects the input dimension *I* into the hidden dimension *H* using fully connected layers with biases. The LSTM has four gates: input, forget, output, and candidate cell. Each gate requires parameters for both the input dimension *I* and the hidden dimension *H*. In an LSTM, these gates collectively account for $8H^{2}+8H$. The decoder then maps the hidden dimension *H* to the output dimension *A*. The actor network parameter count can then be expressed as follows:

(20) $$\begin{array}{c} N_{\mathrm{Actor}}=\underset{\mathrm{Encoder}}{\left(\underset{︸}{H\times I+H}\right)}\;+\;\underset{\mathrm{LSTM}}{\left(\underset{︸}{8H^{2}+8H}\right)}\;+\;\underset{\mathrm{Decoder}}{\left(\underset{︸}{H\times A+A}\right)}\;+\;\underset{\mathrm{LayerNorm}}{\left(\underset{︸}{2H}\right)}. \end{array}$$

Similarly, for a critic network that receives connected inputs of states and actions, which have the dimension of (I+A), the total number of parameters can be expressed as follows:

(21) $$\begin{array}{c} N_{\mathrm{Critic}}=\underset{\mathrm{Encoder}}{\left(\underset{︸}{H\times (I+A)+H}\right)}\;+\;\underset{\mathrm{LSTM}}{\left(\underset{︸}{8H^{2}+8H}\right)}\;+\;\underset{\mathrm{Decoder}}{\left(\underset{︸}{H\times A+A}\right)}\;+\;\underset{\mathrm{LayerNorm}}{\left(\underset{︸}{2H}\right)}. \end{array}$$

The number of parameters of actor and critic networks in (20) and (21) quantify the model complexity and the computational requirements for training the networks. The procedure of the proposed scheme is summarized in Algorithm 1. The training of the proposed network begins by initializing the parameters for the actor and centralized critic networks, along with their corresponding target networks (lines 1–2). An empty replay buffer *D* is created to store state, action, and reward tuples (line 3). The main training loop proceeds over a specified number of episodes and time steps (lines 4–6). At each time step *t*, each agent observes local state $s_{k}^{t}$ and selects an action $a_{k}^{t}$ based on the policy of its actor network (lines 7–11). These actions are then executed jointly in the environment, which returns the next states and corresponding rewards ($r_{1}^{t},\ldots ,r_{K}^{t}$) for all agents (lines 12). The resulting experience tuples ($s^{t},a^{t},r^{t},s^{t+1}$) are stored in the replay buffer (line 13). A mini-batch of data is randomly sampled from *D* (lines 15–16) and used to update the critic network by computing target Q-values and minimizing a loss function (lines 18–22). The actor network of each agent is then updated by maximizing the Q-value output of the critic with respect to its local actions. Finally, the parameters of the main (online) networks are softly updated to the target networks using a soft update parameter τ (lines 23–34), ensuring training stability by gradually reflecting the newly learned parameters in the target networks.
**Algorithm 1** The training of the proposed network with *K* agents.  1:Initialize the parameters of the actor networks, $\left[\theta_{1},\ldots ,\theta_{K}\right]$, and the parameter of the centralized critic network, ϕ;  2:Initialize the parameters of the target actor networks, $\left[\theta_{1}^{\prime },\ldots ,\theta_{K}^{\prime }\right]$ and the parameter of the centralized target critic network, $\phi^{\prime }$;  3:Generate experience replay buffer *D*;  4:**for** each episode *e* **do**  5:      Initialize environment;  6:      **for** time step t=1 to *T* **do**  7:          Get state $s_{1}^{0},\ldots ,s_{K}^{0}$ from environment;  8:          **for** each agent k=1 to *K* **do**  9:                Select action $a_{k}^{t}=\mu_{\theta_{k}}\left(s_{k}^{t}\right)$;10:          **end for**11:          Execute joint actions $a^{t}=\left(a_{1}^{t},\ldots ,a_{K}^{t}\right)$;12:          Get next states $s_{1}^{t+1},\ldots ,s_{K}^{t+1}$ from the environment and rewards $r_{1}^{t},\ldots ,r_{K}^{t}$;13:          Store the experience tuple $\left(s^{t},a^{t},r^{t},s^{t+1}\right)$ in *D*;14:      **end for**15:      **if** *D* is sufficiently large **then**16:            Sample a random mini-batch *B* from *D*;17:            **for** each sample b∈B **do**18:                  **for** each agent k=1 to *K* **do**19:                        Compute the target action $a_{k}^{\prime }=\mu_{\theta_{k}^{\prime }}\left(s_{k}^{\prime }\right)$ for the next state $s_{k}^{\prime }$;20:                  **end for**21:                  Compute the target Q-value for each agent in Equation (15);22:            **end for**23:            Update the critic network by minimizing the loss in Equation (16);24:            Perform a gradient descent step on ϕ using $\nabla_{\phi }L\left(\phi \right)$ in Equation (17);25:            **for** each agent k=1 to *K* **do**26:                  Update the actor network parameters $\theta_{k}$ in Equation (18);27:            **end for**28:            Soft-update the target networks in (19);29:      **end if**30:**end for**

## 5. Simulation Results

### 5.1. Simulation Environment

**Figure 5:**
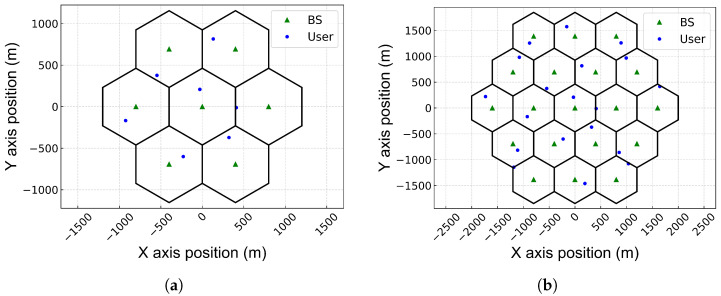
Multi-cell network layout. (**a**) A 7-cell network.; (**b**) A 19-cell network..

We evaluate the performance of the proposed power control scheme based on the LSTM-based MAAC network in hexagonal 7- or 19-cell networks, where each BS serves one user, i.e., there is one user per cell. We assume a hexagonal network topology because the hexagonal grid has been widely adopted in various fields due to its tiling efficiency. Moreover, we employ a 7-cell or 19-cell network topology because most of the conventional power control studies have considered these configurations [19,34]. The proposed MADRL network can be extended to other configurations, such as a triangular 6-cell layout or alternative geometries. However, the hexagonal layout is generally preferred over a triangular layout in cellular networks due to its uniform coverage, equal distance between cells, efficient frequency reuse, geometric properties, and simplicity in network planning. As the network topology expands, the number of agents increases, making DQN-based methods that require signaling between agents less advantageous. Consequently, methods without signaling exchanges between agents, such as QMIX or the proposed scheme, are superior. Furthermore, as the network topology grows, the simulation time also increases. Each BS is positioned at the center of its cell, where the distance between BSs is 800 m. Users are randomly distributed within the cell, but they are assumed to be located at least 100 m away from the BS. We assume that the link between a BS and a user remains during the simulation. Figure 5 illustrates the layout of 7- or 19-cell networks.

The maximum transmit power of each BS is 30 dBm, and the AWGN power is set to −114 dBm over a 10 MHz channel. The path loss between a BS and a user is modeled according to 3GPP TR 25.942, i.e., the path loss is given by $128.1+37.6Log_{10}\left(d\right)$ (in dB), where *d* is the BS-to-user distance in km. The simulation parameters used in this paper are summarized in Table 2.

We set the replay buffer size $\left|D\right|$ and the mini-batch size $\left|B\right|$ to be 1000 and 32, respectively. To ensure that each episode is an independent learning process, we reset the agent’s channel state at the end of each episode. The parameters for training the proposed network are shown in Table 3.

We compared the performance of the proposed scheme with those of five schemes: FP, DQN-based MADRL, QMIX-based MADRL, maximum power, and random power schemes. First, the FP scheme is a centralized scheme, and it requires immediate global CSI from all receivers [27]. Hence, the FP scheme is an ideal technique that is not practical because obtaining immediate CSI from all receivers is difficult. For the FP scheme, we ran up to 100 iterations to determine the transmit power of each time slot. Second, the DQN-based MADRL scheme uses a single meta-agent approach to share the DQN network parameters (weights and biases) among agents [34]. This approach can form consistent behavior patterns in the entire system because all agents follow the same policy, but it may entail additional signaling overhead and computational complexity because the DQN network parameters are shared among the agents even during the inference phase. Third, the QMIX-based MADRL scheme estimates the global Q-value by combining the local Q-values of each individual agent [19]. Additionally, we consider a maximum power scheme, where all BSs use the maximum transmit power, and a random power scheme, where each BS randomly selects its transmit power.

### 5.2. Signaling Overhead Due to Information Exchanges Between Agents

In a multi-cell network, it is highly impractical for an agent to obtain instantaneous global CSI of desired and interfering links. Moreover, exchanging information between agents to obtain the CSI of neighboring agents incurs signaling overhead and hinders scalability according to the network size. The DQN-based MADRL scheme of [34] collects both global and local CSI from neighboring agents, nearby BSs, and BSs connected via desired links while considering time delays. Hence, the DQN-based MADRL scheme incurs significant signaling overhead. In contrast, the QMIX-based MADRL scheme collects only local CSI, and it uses three defined states per agent [19]. Similarly, the proposed scheme also collects only local CSI, and it uses only two defined states per agent, which is the least amount of CSI required.

The signaling overhead depends on the number of signaling messages exchanged between agents and the number of parameters per signaling message, during each time slot. The DQN-based scheme of [34] requires information from other agents, which causes signaling message exchanges. In contrast, the QMIX-based and proposed schemes use the local information of each agent, so there is no signaling message exchange exchange. In the DQN-based scheme of [34], the defined states of each agent include 57 parameters, 50 of which are received from neighboring agents [19]. Consequently, the signaling overhead in the DQN-based scheme is approximately $50N_{b}$ bits, and if 32-bit single precision is used, $N_{b}$ equals 32 bits [42].

### 5.3. Average Data Rate Performance

To evaluate the performance of the power control schemes, we use the metrics, “moving average data rate per link” and “empirical cumulative distribution function (CDF).” The moving average data rate, representing the data rate averaged over 300 time slots, is one of the major performance metrics because it indicates cell capacity in terms of data rate. Therefore, we plot the moving average data rate over time and the probability distribution of the moving average data rate for the entire simulation duration.

**Figure 6:**
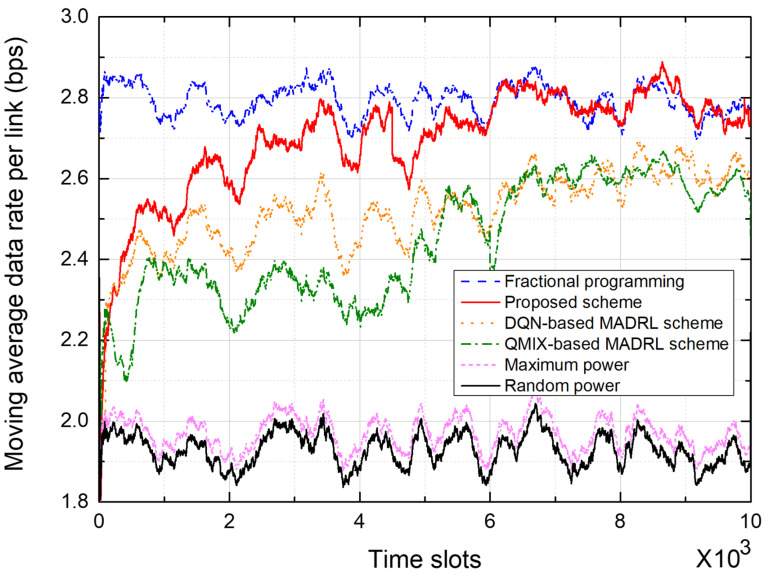
Moving average data rate in a 7-cell network.

**Figure 7:**
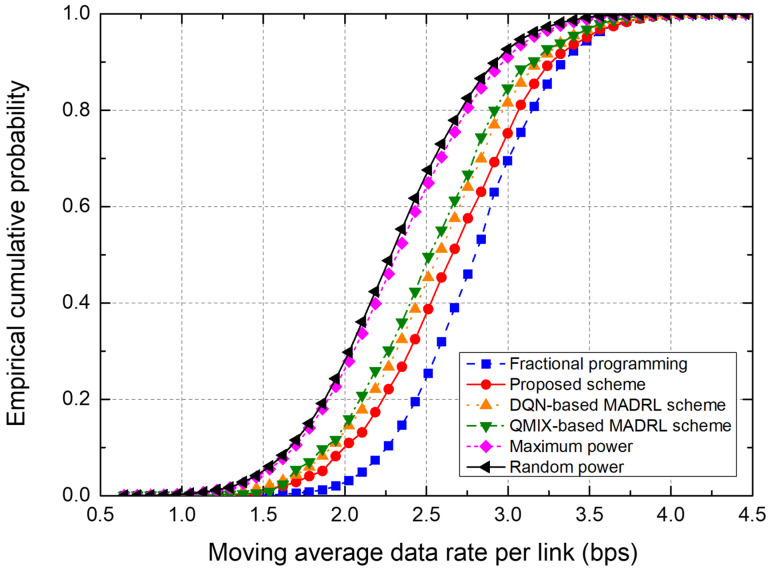
Empirical CDF of the moving average data rate in a 7-cell network.

Figure 6 and Figure 7 show the performance of power control schemes in the 7-cell networks. Figure 6 shows the moving average data rate per link during the training of a network. In the MADRL-based power control schemes, the data rate performance initially fluctuates somewhat but gradually stabilizes and converges. In particular, the proposed scheme outperforms conventional DQN-based and QMIX-based schemes; furthermore, the performance of the proposed scheme converges quickly. Over the last 200 time slots, the proposed scheme outperforms the DQN-based scheme by about 4.76% and the QMIX-based scheme by about 6.55%, in terms of the moving average data rate.

Figure 7 shows the empirical CDF of the moving average data rate from the test on the 7-cell network. The FP scheme shows the best performance because the transmit powers of all BSs are controlled in a centralized manner on the basis of the instantaneous CSI from all receivers. The proposed scheme outperforms conventional DQN- and QMIX-based schemes. Note that the proposed scheme uses only local CSI at each agent, which significantly reduces the signaling overhead by eliminating information exchange between agents. In particular, the DQN-based MADRL scheme requires information exchange between agents, thus incurring significant signaling overhead.

**Figure 8:**
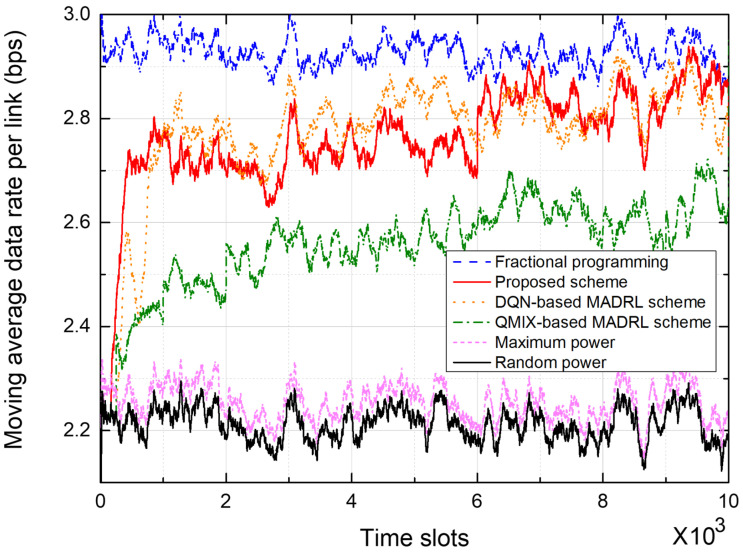
Moving average data rate in a 19-cell network.

**Figure 9:**
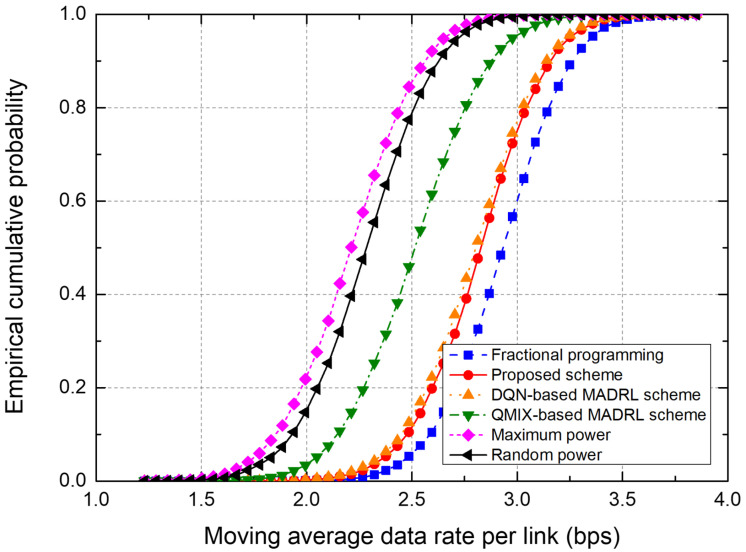
Empirical CDF of the moving average data rate in a 19-cell network.

Figure 8 and Figure 9 show the performance of power control schemes in 19-cell networks. Figure 8 shows the moving average data rate per link during the training of a network. Over the last 200 time slots, the proposed scheme outperforms the DQN-based scheme by about 3.46% and the QMIX-based scheme by about 9.14%, in terms of the moving average data rate. In particular, while the DQN-based MADRL scheme shows a slow convergence rate in a 7-cell network, as shown in Figure 6, it shows a similar convergence rate to the proposed scheme in a 19-cell network. In a DQN-based MADRL network, as the number of agents increases, various channel datasets can be learned from more neighboring agents, and therefore, it has a fast convergence rate due to the properties of the DQN depending on the size of the channel dataset. In the DQN-based scheme, parameter sharing is more prevalent in the 19-cell network compared to the 7-cell network, which results in improved performance in the 19-cell network because a single centralized model can comprehensively learn the wireless channels [34]. However, notice that the DQN-based scheme requires the signaling messages to exchange information between agents, which causes a significant increase in signaling overhead depending on the number of agents.

Figure 9 shows the empirical CDF of the moving average data rate from the test on the 19-cell network. The FP scheme shows the best performance thanks to the centralized control of the transmit power. The proposed and DQN-based schemes show similar performance, although the proposed scheme uses only local CSI at each agent. Moreover, the QMIX-based MADRL scheme shows relatively much lower performance than the proposed and DQN-based schemes.

As the network topology expands, the number of agents increases, which in turn raises the number of states and complicates the interference patterns, leading to an increase in convergence time. Figure 6 and Figure 8 show that the convergence time increased by approximately 15–20% in a 19-cell network compared to a 7-cell network. We can anticipate a similar linear or mildly polynomial increase in larger networks. However, because the LSTM in the proposed scheme effectively compresses and retains relevant past sequence information in its hidden state, the proposed scheme is expected to show improved convergence speed [24].

In Figure 6, Figure 7, Figure 8 and Figure 9, the proposed scheme outperforms the conventional QMIX-based scheme of [19]. Both CTDE and QMIX utilize only the agents’ instantaneous observations and actions at the current time slot as inputs. Consequently, they cannot capture the temporal variability of the wireless channel or any history of past power-control decisions, making them overly sensitive to transient noise or interference spikes and prone to unstable policy convergence. Furthermore, QMIX’s mixing network combines only single-time-step Q-values in a monotonic manner, which means it cannot account for dynamic changes in agent interactions or any history of those interactions. In contrast, the proposed network incorporates the LSTM to continuously track state changes over time and preserve historical observation data, where the state consists of local CSI and neighbor observations. Hence, the proposed LSTM-based MAAC network can make decisions based not only on the current channel state but also on historical interference patterns and previous power-control actions. Consequently, embedding an LSTM-based sequence model within each agent effectively overcomes the lack of temporal modeling and partial observability issues found in CTDE and QMIX.

### 5.4. Complexity Performance

We evaluate the complexity of the proposed scheme in terms of space and time, in comparison with the DQN- and QMIX-based MADRL schemes. The space complexity, which is the amount of memory used for high-dimensional problems, can be represented by the number of parameters used; moreover, the time complexity can be represented by the number of multiply–accumulate (MAC) operations.

In the DQN-based scheme, a fully connected network is used per agent, consisting of an input layer with 57 neurons, an output layer with 10 neurons, and three hidden layers with 200, 100, and 40 neurons, respectively. The QMIX-based scheme has a decision network consisting of one actor network and two critic networks, a mixing network, and a hypernetwork. Here, each subnetwork of the decision network has an input layer with three neurons, an output layer with a single neuron, and three hidden layers with 128 neurons each. The mixing network and hypernetwork also use a fully connected layer of 128 neurons to generate a global Q-value and corresponding weights and biases.

Table 4 summarizes the number of parameters and the number of MAC operations obtained by profiling the DQN-based, QMX-based, and proposed schemes using the “thop.profile” library of PyTorch version 1.13.1 [43]. The proposed scheme increases the complexity compared with the DQN-based scheme, but significantly reduces the space and time complexity compared with the QMX-based scheme. In particular, compared with the QMIX-based scheme, the proposed scheme reduces the number of parameters by about 77.6% and the number of MAC operations by about 38.8%, in a 7-cell network. Compared with the DQN-based scheme, the proposed scheme increases the complexity, but it should be noted that the DQN-based scheme increases the signaling overhead according to the number of agents.

## 6. Conclusions

In multi-cell networks, controlling the transmit power of each BS is highly important to mitigate inter-cell interference and maximize the overall cell capacity. In this paper, we developed a novel transmit power control scheme that leverages a MADRL approach within the framework of CTDE. In particular, the proposed scheme uses only local CSI at each agent without exchanging information between agents, which significantly reduces the signaling overhead between agents. The proposed MADRL network is based on an actor–critic network and incorporates an LSTM architecture to process local CSI at each agent, thereby allowing the algorithm to dynamically adapt to variations in the wireless channel over time. Moreover, by processing local CSI through an LSTM network, the proposed method is able to retain state information over multiple time slots, allowing for more informed and adaptive decision-making in the face of non-stationary channel conditions. The proposed LSTM-based MAAC network outperforms the conventional DQN-based or QMIX-based MADRL networks in terms of the average data rate of the entire network. Moreover, the proposed network uses only two states in each agent, which makes convergence faster. Furthermore, by using the CTDE framework, our approach benefits from robust centralized training while retaining the scalability and practicality of decentralized execution. This combination enables our solution to effectively capture the complex temporal and spatial dynamics present in multi-cell networks.

When applying the proposed LSTM-based MAAC network to practical systems, it is important to consider the inference latency and its integration with existing RRM systems into account. First, the inference time cannot be non-negligible depending on the BS hardware. Although the proposed scheme significantly reduces the space and time complexity compared with the QMIX-based scheme. Therefore, by quantizing the LSTM network from 32-bit floating point to 8-bit integers depending on the hardware specifications, we can reduce both the required memory and computational complexity while sacrificing some performance. Second, it may be difficult to integrate the proposed machine learning-based technology into existing RRM systems due to vendor-specific implementations. However, 3GPP has recently introduced machine learning techniques in RRM, and the open radio access network (O-RAN) alliance has also adopted machine learning techniques [44]. Therefore, the proposed distributed power control scheme is expected to be applicable to 6G systems.

Future research may include learning models such as federated training or distributed training to reduce centralized training overhead, and practical environments such as multi-cell, multi-user networks under dynamic user mobility. First, federated learning or distributed learning models can be studied to reduce centralized learning overhead. Generally, distributed learning is about making centralized training faster, whereas federated learning is about enabling collaborative training. The choice between federated and distributed learning depends on specific requirements related to data privacy, resource availability, and the scale of the data and computation involved. In particular, the signaling overhead exchanged between nodes should be accounted for. Second, future research could consider user selection, scheduling, and power control simultaneously in a multi-cell multi-user network that serves multiple users per cell, with users moving at different speeds.

## Figures and Tables

**Table 1:** Symbols and descriptions.

Symbol	Description
K	The set of BS indices
*K*	The number of BSs
$u_{k}$	The user served by BS *k* in time slot *t*
$g_{k,k}^{t}$	The downlink channel gain from BS *k* to user $u_{k}$ in time slot *t*
$h_{k,k}^{t}$	The small-scale fading from BS *k* to user $u_{k}$ in time slot *t*
$e_{k,k}$	The channel innovation process from BS *k* to user $u_{k}$ in time slot *t*
ρ	The correlation coefficient
$f_{d}$	The maximum Doppler frequency (Hz)
*T*	The slot duration
$\beta_{k,k}^{t}$	The large-scale fading component from BS *k* to user $u_{k}$ in time slot *t*
$X_{\alpha }^{t}$	The shadow-fading component in time slot *t* (dB)
$\sigma_{s}$	The shadowing standard deviation (dB)
$\sigma^{2}$	The additive white Gaussian noise (AWGN) power spectral density
$\sigma_{k,k}^{\mathrm{shad}}$	The shadow channel innovation process from BS *k* to user $u_{k}$
$C_{k}^{t}\left(p\right)$	The received SINR of user *k* at time slot *t* (dB)
$p^{t}$	The downlink transmit power set in time slot *t*
$p_{k}^{t}$	The transmit power of BS *k* in time slot *t* (dBm)

**Table 2:** Simulation parameters.

Parameter	Value
Number of cells	7,19
Maximum transmit power	30 dBm
Pathloss model	3GPP TR 25.942
Slot duration, *T*	20 ms
Number of slots in simulation	10,000 slots
Carrier frequency	2 GHz
Frequency bandwidth	10 MHz
Shadowing distribution, $\sigma_{s}$	Log-normal, 8 dB
Maximum Doppler frequency, $f_{d}$	10 Hz
Noise power spectral density, $\sigma^{2}$	−114 dBm

**Table 3:** Proposed scheme parameters.

Parameter	Value
Number of training episodes	100
Number of neurons in the encoder	128
Number of neurons in the actor LSTM	32
Number of neurons in the critic LSTM	64
Number of neurons in the decoder	128
Actor optimizer	Adam
Critic optimizer	Adam
Actor learning rate, α	0.0052
Critic learning rate, β	0.015
Soft update parameter, τ	0.1
Reward discount factor, γ	0.95
Loss function	Clipped MSE

**Table 4:** Complexity comparison.

Model	7-Cell Network		19-Cell Network	
	# of Parameters	# of MACs	# of Parameters	# of MACs
DQN-based scheme	36,150	250,600	36,150	680,200
QMIX-based scheme	931,990	927,744	2,358,970	2,348,544
Proposed scheme	208,424	567,392	464,564	1,540,064

## Data Availability

Data is contained within the article.
